# Cardiac Biomarkers and the Diagnosis of Myocardial Infarction in Women

**DOI:** 10.1007/s11886-017-0839-9

**Published:** 2017-04-08

**Authors:** Anoop S. V. Shah, Amy V. Ferry, Nicholas L. Mills

**Affiliations:** BHF Centre for Cardiovascular Science, University of Edinburgh, Edinburgh, EH16 4SB UK

**Keywords:** Sex, Cardiac troponin, Biomarkers, Myocardial infarction

## Abstract

**Purpose of review:**

Women with suspected acute coronary syndrome are less likely to undergo investigation or receive treatment than men, and women consistently have poorer outcomes. This review summarises how the latest development in cardiac biomarkers could improve both diagnosis and outcomes in women.

**Recent findings:**

Novel high-sensitivity cardiac troponin assays have identified differences in the reference range and therefore diagnostic threshold for myocardial infarction in men and women. These differences are present across multiple populations with different ethnic backgrounds and for a range of assays. The use of a uniform threshold for cardiac troponin does not provide equivalent prediction in men and women, with lower thresholds needed for women to provide comparable risk stratification.

**Summary:**

Sex differences in cardiac troponin concentrations are not widely recognised in clinical practice and may be contributing to the under-diagnosis of myocardial infarction in women and discrepancies in patient care and outcomes.

## Introduction

In September 2000, the United Nations Millennium Declaration committed to promote gender equality as one of their primary goals to achieve health equity. Globally, coronary heart disease remains the major cause of death in women in both high-and low- or middle-income nations [1]. Despite this, sex differences remain in multiple aspects of cardiovascular care including diagnosis, access to investigation and treatment and outcomes [2••]. Furthermore, these differences are prevalent even in highly developed health-care systems such as the USA [2••, 3]. The most recent scientific statement from the American Heart Association (AHA) highlights two key areas that contribute to sex differences: biological or social factors, and the underrepresentation of women in clinical trials [4].

Cardiovascular medicine is fortunate to have a high-quality evidence base derived from multiple randomised control trials to guide our practice. However, a comprehensive summary by Nanette Wenger highlights how medical research has neglected the health needs of women especially in cardiovascular medicine [2••, 4]. Whilst improvements have been made to increase recruitment of women in clinical trials, to date, large randomised control trials include a majority of men. As such, the evidence on safety and efficacy of key therapeutic interventions in acute myocardial infarction are limited in women with data largely derived from their male counterparts.

**Fig. 1:**
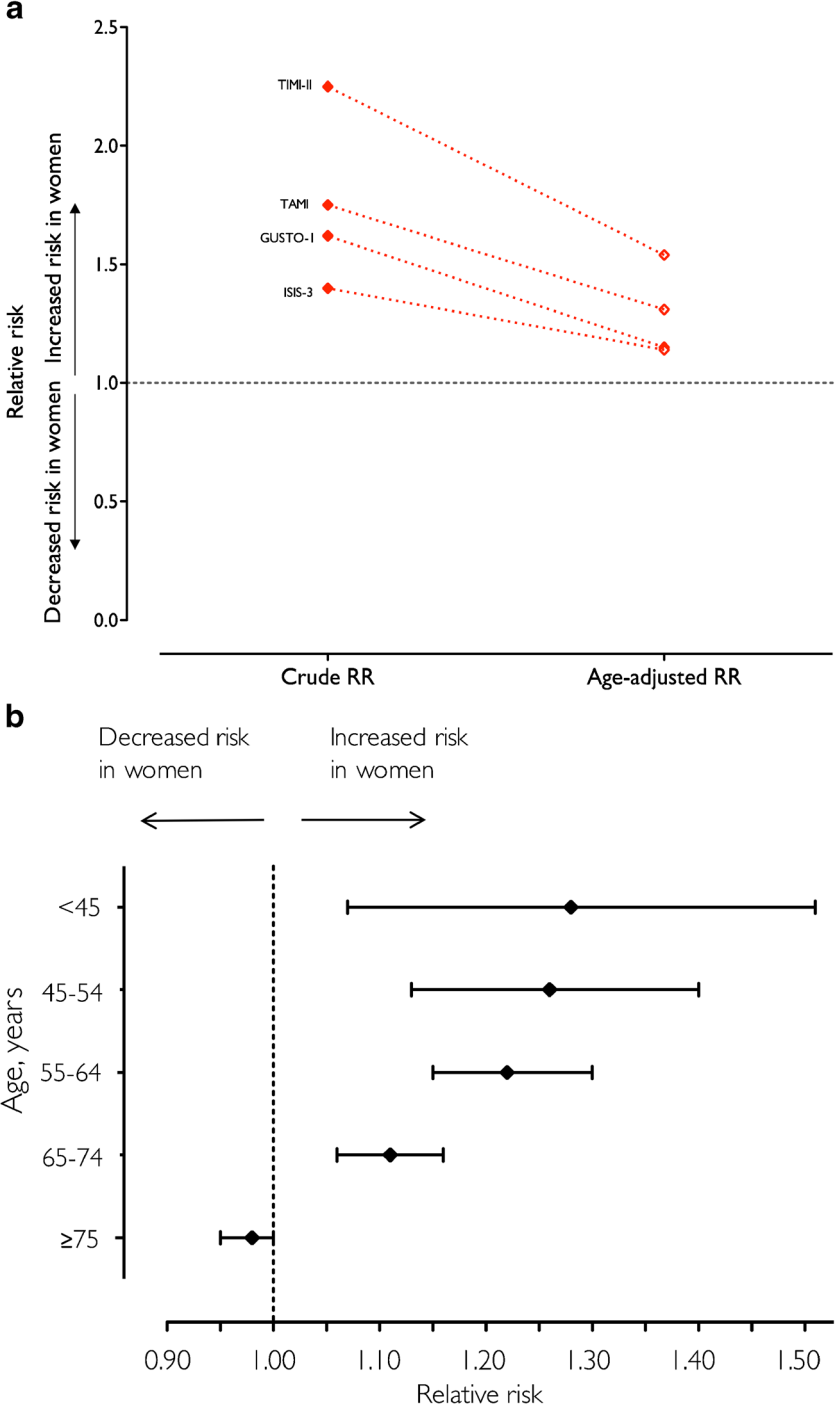
Risk of mortality in women compared to men in randomised control trials before and after adjustment for age (**a**) and stratified by age in a large cohort study (**b**). *TIMI II* Thrombolysis in myocardial infarction II [6], *GUSTO* Global Utilization of t-PA and Streptokinase for Occluded Coronary Arteries (GUSTO-I) trial [7], *TAMI* Thrombolysis and Angioplasty in Myocardial Infarction [8], *ISIS-3* Third International Study of Infarct Survival [9]

More concerning is the evidence from epidemiological studies and randomised controlled trials of disparities in outcomes following myocardial infarction in men and women [3, 5]. The AHA scientific statement on myocardial infarction in women highlighted this issue [4]. In data derived from large multi-centred randomised control trials, women consistently have higher case fatality rates compared to men, and these differences exist despite adjusting for age and comorbid conditions (Fig. 1a). Indeed, it is in the younger age groups that women are more likely than men to have an adverse event following myocardial infarction (Fig. 1b). In a consecutive series of over a million patients with acute coronary syndrome, even women <45 years were at increased risk of death compared to men of a similar age (relative risk [RR] 1.3 [95% confidence interval 1.2 to 1.4]) [3]. It is less clear what biological or social factors are responsible for these disparities. Many have been proposed, including sex differences in the pathobiology of acute myocardial infarction, in the time taken to seek medical advice following the onset of symptoms and in the efficacy of treatment for myocardial infarction. There may also be differences in the presenting symptomology between men and women, which may result in misdiagnosis, delay in providing evidence-based therapy and explain the worse outcomes seen in women.

An alternative explanation, and one that is gaining increasing attention, is that we are under-diagnosing myocardial infarction in women by not recognizing that there are important differences in the reference range and diagnostic threshold of cardiac biomarkers between men and women.

### Diagnosis of Myocardial Infarction

The diagnosis of myocardial infarction is based on symptoms or signs of myocardial ischaemia and biomarker evidence of myocardial necrosis. The definition is largely unchanged from the initial MONICA World Health Organization (WHO) statement. What has, however, changed significantly over the last few decades is our ability to detect and quantify myocardial necrosis. The aim of the original WHO definition was to standardise diagnosis and permit monitoring of trends and determinants of myocardial infarction in epidemiological studies. This definition was not considered sufficiently specific in clinical practice for the assessment of individual patients and was open to interpretation [10]. The need for a more precise definition of myocardial infarction coupled with the availability of more sensitive and specific biomarkers of myocardial necrosis resulted in a series of consensus statements and revisions of the definition of myocardial infarction.

Serum biomarkers have been used to assist in the diagnosis of acute myocardial infarction for over half a century with aspartate transaminases in the 1950s and lactate dehydrogenase in the 1970s [11]. However, it was not until the late 1970s when an isoenzyme of creatine kinase (CK-MB) was discovered that a biomarker revolutionised the clinical diagnosis of myocardial infarction. In 2003, Apple et al. evaluated seven CK-MB mass assays in 696 healthy participants, demonstrating that the 99th centile upper reference limit was two to threefold higher in men [12]. As such, the National Academy of Clinical Biochemistry guidelines advocated the use of sex-specific 99th centile upper reference limits when using CK-MB with a class 1C recommendation [13].

### Cardiac Troponins

Troponin is a 3-piece regulatory protein complex present in cardiac and skeletal muscle and is integral to muscle contraction. Both cardiac troponin T and troponin I are derived from genes specific to the myocardium [14, 15], whereas troponin C is present in both cardiac and skeletal muscles with no specific isoform for cardiac tissue [14].

Given the high specificity of cardiac troponin T and troponin I to the myocardium, quantification of these biomarkers has now become integral to the diagnosis of myocardial infarction. The latest consensus statement defines myocardial infarction as a rise and/or fall in cardiac troponin with at least one value above the 99th centile upper reference limit in the context of symptoms or clinical evidence of myocardial ischaemia [16]. This was a paradigm shift replacing CK-MB as the gold standard for the diagnosis of myocardial infarction.

Over the last decade, troponin assays have become increasingly sensitive, from the older generation conventional assays to the more sensitive contemporary troponin assays. The latest developments in assay technology have led to the increasing use of high-sensitivity troponin assays. These assays have been defined as high-sensitivity by the International Federation of Clinical Chemistry Task Force based on assay performance [17]. A high-sensitive assay should meet two basic criteria: first, the total coefficient of variation (a measure of [im]precision) should be ≤10% at the 99th centile of a healthy reference population and, second, at least 50% (and ideally 90%) of the reference population should have a measurable troponin concentration above the limit of detection.

### Sex Differences in Cardiac Troponin Concentrations

The older generation cardiac troponin assays had initially been thought to be good gender-independent markers for diagnosis in acute coronary syndrome. Key papers published in the last two decades challenged this view. In the 1990s, Hamm et al. measured cardiac troponin T in men and women with unstable angina in whom acute myocardial infarction was excluded. The study showed that troponin T concentrations were measurable (>200 ng/L) in 43% of men and only 27% of women [18]. This initial observation was supported by Wiviott et al. who demonstrated that in patients with unstable angina or non-ST segment elevation myocardial infarction pooled from four randomised control trials, men were more likely to have elevated cardiac biomarkers including cardiac troponin I, cardiac troponin T and CK-MB compared to women using older generation assays with a single diagnostic threshold [19].

More recently, developments in assay technology have greatly enhanced sensitivity and, for the first time, have been able to quantify circulating troponin in the majority of individuals in a normal healthy reference population [20]. Measuring troponin using high-sensitivity assays has revealed important differences between men and women, with the 99th centile reference limits up to two-fold higher in men [20]. This observation has been consistent across all troponin assays that have been evaluated and has now been reported in multiple populations from different ethnic backgrounds (Table 1). Indeed, the reference range has been studied for 19 different assays in the same population, demonstrating higher 99th centile upper reference limits for the two clinically available high-sensitivity assays: cardiac troponin T (20 ng/L men, 13 ng/L women) and cardiac troponin I (36 ng/L men, 15 ng/L women) [20].

**Table 1:** Studies reporting 99th centile values for high-sensitivity cardiac troponin T and cardiac troponin I in healthy men and women

Troponin assay	Manufacturer	Author and year	Population	Age (range)	Sex, females (%)	99th Centile		Region	Defining healthy reference populations		
						Males	Females		History and medical records	Biomarkers	Cardiac imaging
Troponin T	Roche	Giannitsis et al. [21]	616	20–71	307 (49.8%)	14.5	10	USA	Yes	No	No
		Saenger et al. [22]	533	20–71	265 (49.7%)	15.5	9	USA, Europe	Yes	No	No
		Koerbin et al. [23]	111	25–74	49 (44.1%)	12.9	11	Australia	Yes	Yes	Yes
		Collinson et al. [24]	545	45–89	286 (52.4%)	22.8	12.8	UK	Yes	Yes	Yes
		Mingels et al. [25]	479	26–71	215 (44.9%)	16	8	USA	Yes	No	No
		Apple et al. [20]	524	18–64	252 (48.0%)	20	13	USA	Yes	No	No
Troponin I	Abbott	Aw et al. [26]	1120	35–65	523 (46.7%)	32.7	17.9	Singapore	Yes	No	No
		Apple et al. [20]	524	18–64	252 (48.0%)	36	15	USA	Yes	No	No
		Koerbin et al. [26]	497	20–84	266 (53.5%)	14	11	Australia	Yes	Yes	No
	Beckman	Apple et al. [20]	524	18–64	252 (48.0%)	52	23	USA	Yes	No	No
	Singulex	Apple et al. [27••]	348	18–76	201 (57.8%)	16.5	9.3	USA	Yes	No	No
	Siemens	Apple et al. [20]	524	18–64	252 (48.0%)	36	30	USA	Yes	No	No
		Apple et al. [20]	524	18–64	252 (48.0%)	81	42	USA	Yes	No	No
		McKie et al. [28]	565	50–61	305 (54.0%)	55	33	USA	Yes	Yes	Yes

**Fig. 2:**
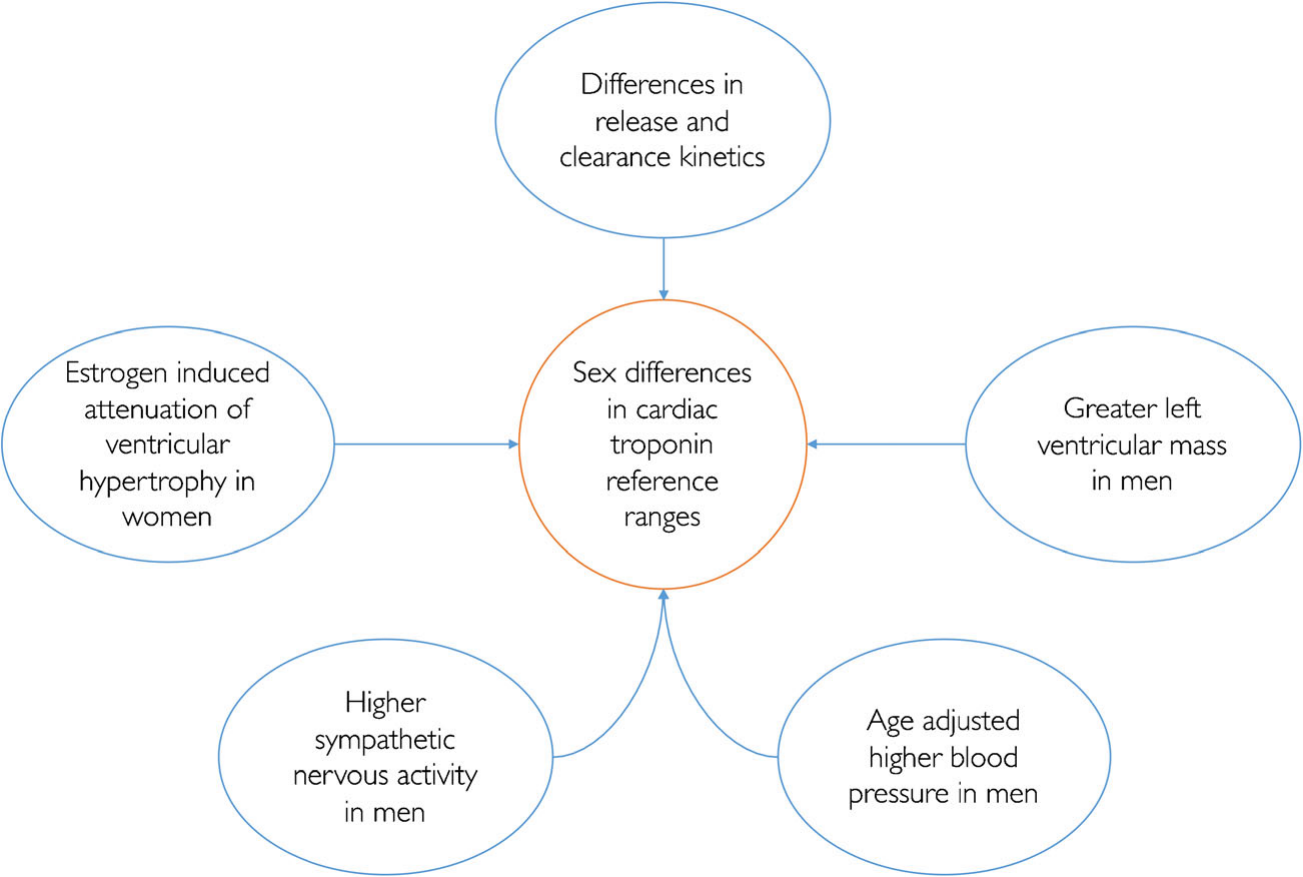
Proposed biological explanations for differences in the distribution of cardiac troponin in men and women

The mechanisms through which cardiac troponin is released into the circulation in the apparent ‘healthy’ state is unclear, but may reflect cardiomyocyte apoptosis and cell turnover, hypertrophy or sub-clinical myocardial fibrosis [29, 30]. Furthermore, it is unclear why there is a difference in the distribution of cardiac troponin concentrations in men and women [31], but multiple hypotheses have been proposed (Fig. 2) [29, 32, 33]. In contrast, sex differences in CK-MB concentrations were attributed to differences in skeletal mass, which contributes 1–2% of circulating CK-MB [31] and is likely to explain higher levels in men. This is not the case for cardiac troponin, which has excellent tissue specificity, and therefore differences in the reference limits are likely to reflect differences in cardiovascular physiology or the prevalence of sub-clinical pathology in men and women.

Likewise, in acute coronary syndrome, women have lower cardiac troponin concentrations than men, perhaps suggesting differences in the mechanism of myocardial infarction between sexes. For example, in women with acute coronary syndrome enrolled in the TACTICS-TIMI 18 trial, the odds of having an elevated plasma cardiac troponin T or I concentrations were 0.53 [95% CI 0.43 to 0.68]) and 0.58 [95% CI 0.46 to 0.73], respectively, compared to men [19]. Changes in reproductive hormones during the menstrual cycle, pregnancy and menopause influence both vasomotor function and endogenous fibrinolysis [34]. Perhaps, as a consequence, women with acute coronary syndrome are less likely to have evidence of plaque rupture (6.6 versus 16.3%; *P* = 0.002) on intravascular coronary imaging studies [35]. Indeed, women are more likely than men to have non-obstructive atherosclerotic disease with either demonstrable vasospasm, spontaneous coronary artery dissection or plaque erosion with microembolization [36]; all of which are likely to result in less myocardial injury and lower cardiac biomarker concentrations.

### Should We Apply Sex-Specific Thresholds for the Diagnosis of Myocardial Infarction?

Both inconvenience and a potential source of confusion for clinicians have been used as arguments against the use of sex-specific thresholds [37]. However, sex-specific reference ranges are not unfamiliar in clinical medicine and clinicians have coped with such scenarios for decades. Common laboratory parameters, including haemoglobin and glomerular filtration rate, account for sex when determining reference limits. Traditionally, clinical decision limits adjusted for sex have been solely based on reference range studies [12], but given the critical role of cardiac troponins in the diagnosis of myocardial infarction, additional evidence is required from outcome studies and from studies that evaluate diagnostic accuracy of sex-specific thresholds.

### Sex Differences in Troponin and Cardiovascular Outcomes

Recently, a number of studies have evaluated differences in the prognostic utility of high-sensitivity cardiac troponin between men and women in stable populations. The Activity and Function in the Elderly study showed that in 1506 participants over the age of 65 years, increasing cardiac troponin T and troponin I concentrations were strongly associated with all-cause mortality. These association varied by sex with women having numerically higher age-adjusted associations with mortality per unit increment in cardiac troponin (log-transformed) compared to men for both troponin T (RR 3.67 [95% confidence interval (CI) 2.31 to 5.81] versus 2.15 [95% CI 1.61 to 2.87]) and troponin I (3.33 [95% CI 2.13 to 5.18] versus 1.92 [95% CI 1.55 to 2.38]) [38]. Similar associations were echoed in the larger HUNT study with 9712 participants including 5281 (54%) women. Increasing high-sensitivity cardiac troponin I concentrations were associated with all-cause mortality (RR 1.17 [95% CI 1.12 to 1.22] per 1 SD increment in log troponin) and cardiovascular death (1.23 [95% CI 1.15 to 1.31]). Again, these associations were significantly and numerically higher in women compared to men for both all-cause mortality (1.33 [95% CI 1.24 to 1.42] versus 1.08 [1.01 to 1.15]) and cardiovascular death (1.44 [95% CI 1.31 to 1.58] versus 1.10 [1.00 to 1.20], *p* value for interaction <0.001) [39]. Eggers et al. observed numerically higher associations between high-sensitivity cardiac troponin I and death or incident cardiovascular disease in women compared to men in 1004 (502 women) elderly persons; however, sex was not a significant effect modifier for either [40].

These studies have important implications for clinical practice in guiding whether to adopt sex-specific thresholds for cardiac troponin. One of the key obstacles, to date, has been the lack of evidence that troponin concentrations above and below sex-specific cut points infer different prognostic information to that of single thresholds. Indeed, if higher troponin levels do infer worse prognosis for women, as some of the larger cohort studies seem to suggest, adopting single troponin thresholds is likely to misclassify some women as low risk with potentially important clinical ramifications.

### Sex Differences in the Diagnosis of Myocardial Infarction

Whilst the Universal Definition of Myocardial Infarction acknowledges that sex-specific differences exist in the upper reference limits for these assays, no firm recommendations for practice were made in the latest iteration [41]. Since the publication of this guideline, several studies have evaluated the impact of sex-specific thresholds using high-sensitivity assays on the diagnosis of myocardial infarction (Table 2) [42••, 43, 44, 46, 47, 48••].

**Table 2:** Studies evaluating the impact of sex-specific thresholds for cardiac troponin on the diagnosis of myocardial infarction

Author and year	Number	Region	Population	Female	Consecutive patients	Study assay	Sex-specific thresholds used	Comments
Shah et al. [42••]	1,126	Scotland	Suspected ACS	504 (45%)	Yes	High sensitivity (Abbott TnI)	M: 34, F:16	Sex-specific thresholds using high-sensitivity assays doubled the diagnosis of myocardial infarction in women
Cullen et al. [43]	2841	Australia and New Zealand	Chest pain	1077 (38%)	No	High sensitivity (Abbott TnI)	M: 34, F:16	Sex-specific thresholds identified women but not men at high risk of MACE at 1 year
Schofer et al. [44]	1560	Germany	Chest pain	541 (35%)	No	High sensitivity (Singulex TnI)	M: 36, F:30	Study showed that using sex-specific thresholds with absolute changes in TnI concentrations performed better than those with relative changes
Mueller-Hennessen et al. [45]	1282	Europe, multi-centre	Suspected ACS	477 (37%)	No	High sensitivity (Roche TnT)	M: 16; F: 9	Increase in the diagnosis of myocardial infarction in women from 16 to 23% but small reduction in the proportion of males diagnosed from 23 to 21%
Gimenez et al. [46]	2734	Europe, multi-centre	Suspected ACS	876 (32%)	No	High sensitivity (Roche TnT)	M: 16; F: 9	Sex-specific thresholds using high-sensitivity assays did not improve diagnosis of myocardial infarction

In a prospective cohort of selected patients with suspected acute coronary syndrome, Mueller-Hennessen et al. reported that age-specific rather than sex-specific thresholds influenced the diagnosis of myocardial infarction using the high-sensitivity cardiac troponin T assay. Across 1282 patients (477 [37%] women) with suspected acute coronary syndrome evaluated using a high-sensitivity troponin T assay, the use of sex-specific compared to uniform thresholds changed the rates of myocardial infarction from 16 to 22% in women and from 23 to 21% in males. However, the use of sex-specific thresholds had minimal impact on risk stratification for death at 1 or 3 months [45]. However, Rubini-Gimenez et al., in 2734 patients (876 [32%] women), did not observe any effect of sex-specific thresholds on the diagnosis of myocardial infarction and concluded that a uniform threshold should remain standard of care for the high-sensitivity cardiac troponin T assay [46].

In contrast, two studies have shown that women with suspected acute coronary syndrome identified by the high-sensitivity cardiac troponin I assay with sex-specific thresholds, but missed with uniform thresholds, are at higher risk of major adverse cardiac events (MACE) at 1 year [42••, 48••]. Cullen et al. demonstrate in a multi-centre cohort study of 2841 patients that MACE was increased in women with troponin concentrations above the sex-specific diagnostic threshold, but below the uniform threshold, and that this association persisted following adjustment for age. Similar findings were reported in a consecutive cohort of 1126 patients with suspected acute coronary syndrome from a single site in Scotland [42••]. This study showed that the use of sex-specific thresholds almost doubled the diagnosis of myocardial infarction in women when compared to a contemporary assay using a uniform threshold.

Finally, it is important to note that there were marked differences in the proportion of women recruited into these cohort studies, and therefore, it is likely that selection bias by gender explains some of the discrepancies observed. The proportion of women included in selected patient cohorts varied from 32 to 38%, but in consecutive patient cohorts, where selection bias is minimised, the proportion of women was higher at 45% (Table 2).

### Methodological Limitations of Studies Evaluating Sex-Specific Thresholds

Unfortunately, the interpretation of diagnostic accuracy studies using high-sensitivity cardiac troponins is challenging. Cardiac troponin is integral to the diagnosis of myocardial infarction, and there is no independent gold standard test to define spontaneous or type 1 myocardial infarction, i.e. myocardial infarction due to plaque rupture and coronary thrombosis [49]. As such, claims that a more sensitive troponin test, whether using uniform or sex-specific thresholds, is better than the reference test may be contested [49, 50••]. The results of these studies are highly influenced by choice of the reference test used to adjudicate the diagnosis. Furthermore, there are important differences between the patients recruited into these cohort studies and those in whom testing is performed in clinical practice. Troponin testing is performed widely in patients attending the Emergency Department, where clinicians are required to rapidly evaluate and exclude multiple potential diagnosis simultaneously. The performance of high-sensitivity cardiac troponin testing is likely to differ here compared to selected patients presenting with symptoms of suspected acute coronary syndrome alone. Women are less likely to be recruited as they are thought to present with less typical symptoms and are older and more likely to have comorbid conditions. As such, selection bias will influence the generalisability of the findings from diagnostic accuracy studies. Due to these limitations, any review of studies comparing diagnostic performance of uniform and sex-specific thresholds requires careful attention to methodology.

When evaluating the use of more sensitive tests and cut points that are lower than those measurable by previous tests, the new test detects additional cases of apparent disease. There will always be uncertainty as to whether those patients identified by the new test should be classified or treated in the same way: Are these additional patients really true positives? This is particularly relevant when retrospectively evaluating the use of sex-specific thresholds as the 99th centile upper reference limit in women is below the uniform threshold used to guide further investigation at the time of presentation. In contrast, in men, the sex-specific 99th centile upper reference limit is higher than the uniform threshold. As such, women identified retrospectively as having elevated cardiac troponin concentrations only when sex-specific thresholds are applied often did not undergo further diagnostic investigations at the time of presentation to inform diagnostic adjudication. Furthermore, women are under-represented in these cohort studies as, unlike their male counterparts, they were less likely to be identified as having an acute coronary syndrome at the time of their presentation.

Glasziou et al. propose an elegant way of addressing this issue: the use of an ‘umpire test’ [50••]. They suggest a three-step process. First, investigators need to highlight cases, which are discordant and identified only by one of the tests. Second, they propose the use of an independent and fair test that is unconditional to either of the tests being evaluated: the umpire test. Third, these ‘umpire tests’ can include a multitude of variables including causal exposures, outcomes and treatment responses [50••]. For example, evidence of myocardial ischaemia on electrocardiography could be used as such an umpire test, where the discrepant group with a higher prevalence of ischaemia would indicate the superior test.

We adapted this methodology in our cohort of patients with suspected acute coronary syndrome [42••]. The analysis showed that those women newly identified as having myocardial infarction using sex-specific thresholds had baseline characteristics, risk factor profiles and prognoses similar to those patients identified as having myocardial infarction using a uniform threshold, and that they were distinct from those identified as not having myocardial infarction using both approaches. Based on a logistic regression model, we showed that those patients reclassified using sex-specific thresholds followed a similar probability distribution and clinical trajectory to those patients classified as myocardial infarction using the uniform threshold [42••]. Importantly, this analysis was conducted in a cohort that included all consecutive patients without selection bias, and therefore, a similar proportion of men and women were evaluated. We conclude that sex-specific thresholds for high-sensitivity cardiac troponin I identify a significant proportion of women missed using uniform thresholds and that these women have other clinical features of myocardial infarction and are at high risk of recurrent events.

### Implementation and Clinical Practice

Lowering the diagnostic threshold following implementation of a more sensitive troponin assay has been shown to improve clinical outcomes [51••]. There was a halving of recurrent myocardial infarction and death in those patients with small previously unrecognised increases in cardiac troponin concentration. These improvements in outcome were associated with better patient care including more specialist referrals, investigations and better provision of evidenced-based therapies [51••]. These observations highlight the importance that clinicians place on an abnormal troponin measurement in guiding clinical decisions and suggest that high-sensitivity troponin assays with even lower diagnostic thresholds have the potential to improve care further.

However, progressively lowering the threshold of plasma troponin concentration in order to define increasing numbers of patients with myocardial infarction is likely to increase sensitivity at the expense of reduced specificity [52]. Elevated cardiac troponin is common in patients without acute coronary syndrome and accounts for up to 30% of all patients with troponin concentrations above the upper reference limit [53, 54]. Furthermore, lowering the diagnostic threshold for cardiac troponin disproportionately increases the detection of patients with elevated cardiac troponin levels in patients without acute coronary syndrome. Outcomes of these patients remain poor after implementation and does not appear to be modifiable [55]. High-sensitivity cardiac troponin assays are likely to increase the frequency of troponin elevations in non-acute coronary syndrome pathologies especially in women if sex-specific thresholds are employed. In women, the proportion with troponin elevations due to non-acute coronary syndrome pathologies increased from 7 to 12%, moving from a less sensitive contemporary troponin assay to a high-sensitivity assay with sex-specific thresholds [42••]. Whether implementation of high-sensitivity cardiac troponin assays and use of sex-specific diagnostic thresholds improve outcomes through better targeting of treatments for coronary heart disease are the focus of an ongoing multi-centre randomised control trial (clinical trials.gov NCT01852123).

## Conclusion

The evidence for and against the use of sex-specific thresholds when implementing high-sensitivity cardiac troponin assays is evolving. There are three main arguments in favour of adopting sex-specific thresholds. First, cardiac troponin concentrations are consistently lower in women than men, across multiple populations from different ethnic backgrounds. Second, cardiac troponin concentrations in stable populations infer different prognostic information in men and women with stronger associations noticed in women when uniform thresholds are applied. It is clear that lower thresholds are needed for women to provide comparable risk stratification. Third, in the setting of suspected acute coronary syndrome, sex-specific thresholds for cardiac troponin I identify women at high risk of future myocardial infarction and death. Finally, it is recognised that women are less likely to receive evidence-based therapy and have worse outcomes in acute coronary syndromes. The use of sex-specific diagnostic criteria may help to address these inequalities. Given these discrepancies in the quality of care provided for men and women, it seems axiomatic that sex-specific criteria be employed in the diagnosis of myocardial infarction.
